# Diversity analysis: Richness versus evenness

**DOI:** 10.1002/ece3.70275

**Published:** 2024-09-23

**Authors:** Tarald O. Kvålseth

**Affiliations:** ^1^ Department of Mechanical Engineering and Department of Industrial and Systems Engineering University of Minnesota Minneapolis Minnesota USA

**Keywords:** biodiversity, diversity, evenness, richness, value validity

## Abstract

Richness and evenness, two important components of diversity, have been the subject of numerous studies exploring their potential dependence or lack thereof. The results have been contradictory and inconclusive, but tending to indicate only a low (positive or negative) correlation. While such reported studies have been based on particular data sets and species abundance distributions, the present article provides the results of a study using randomly generated abundance distributions and hence more generalizable findings and valid statistical results. The results reveal no statistically significant correlation between richness and evenness based on such random sample of abundance distributions and on four well‐known measures of diversity, including Simpson's indices and the entropy index. Of the two diversity components, evenness is found to have the strongest influence on diversity, but for numbers‐equivalent or effective‐number formulations, richness tends to be the most influential diversity component. For analyzing the tradeoff between richness and evenness for any given diversity measure and abundance distribution, the *richness‐evenness curve* is introduced as a new tool for diversity analysis.

## Graphical abstract

Richness and evenness are two important components of any measure of diversity. This article presents a simple graphical method showing the tradeoff between those two components of a diversity measure. Real biological data are used as an illustration.

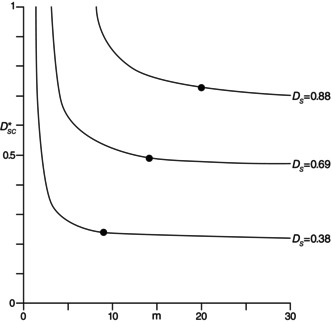

## 1 INTRODUCTION

Diversity is generally considered to consist of two components: *richness* and *evenness*. In biology and ecology, richness typically means the number of different species in a sample or population while evenness refers to the extent to which the different species are equally represented in the sample (population). Diversity increases as the number of species increases and as their relative proportions become increasingly equal or uniform. As stated by Magurran (2004, p. 9), “Species richness is simply the number of species in the unit of study” while “evenness describes the variability in species abundances.” A *diversity index* or measure is a statistic that incorporates information about both richness and evenness.

For a sample, collection, or unit of study with a relative abundance distribution $P_{n}=\left(p_{1},p_{2},\ldots ,p_{n}\right)$ where $p_{i}$ is the proportion (sample probability) of the *i*‐th species and all $p_{i}$'s sum to 1 (i.e., $\sum_{i=1}^{n}p_{i}=1$), the *species richness* is simply defined as *n*. The *species evenness* refers to how evenly (uniformly) the $p_{i}$'s are distributed, but its measurement is not so simple and remains unsettled (see, for example, Kvålseth, 2015; Smith & Wilson, 1996). With diversity being considered a combination of richness and evenness, a wide variety of diversity indices have been proposed over the years (e.g., Daly et al., 2018; Magurran, 2004, ch. 4–5). In spite of such a variety of ways to measure and interpret diversity, it certainly would be informative to explore whether any kind of relationship exists between the richness and evenness components. Such information could provide for a better understanding of diversity as a concept and of its measurement.

In fact, the relationship between richness and evenness, if any, has been the subject of considerable interest, controversy, and numerous studies and publications. Some have emphasized that richness and evenness should be independent components (e.g., Heip, 1974; Peet, 1974: Smith & Wilson, 1996) while Jost (2010) has argued that they cannot possibly be independent. Others have studied the potential richness‐evenness relationship and generally found limited interaction or low correlations between evenness and richness (e.g., Blowes et al., 2022; Bock et al., 2007; Buzas & Hayek, 2005; Gosselin, 2006; Liu et al., 2023; Ma, 2005; Stirling & Wilsey, 2001; Yan et al., 2023; Zhang et al., 2012). Soininen et al. (2012) conducted a meta‐analysis of various studies and found that “significant correlations of species richness and evenness only existed in 71 out of 229 datasets. Eighty‐nine were negative and 140 were positive (p. 803).” In a study by Su (2018), the indication is that any richness‐evenness relationship will depend on the form of the relative abundance distribution.

The various reported studies exploring a potential relationship between richness and evenness have been based on data collected from particular ecological systems and sites. Ma (2005), for example, studied plant species in different field quadrats in Finland as basis for relative abundance distributions. As another example, Su (2018) used sample data for island birds, stream fishes, and zooplankton in specific locations. In all of these studies, not only did the richness and evenness components vary, but the values of the diversity indices also varied. Another complicating factor in understanding the relationship between richness and evenness is that different measures of diversity and of evenness were used in different studies.

Although the results from individual studies of a potential richness‐evenness relationship or dependence are restricted to the particular ecological systems and species abundance distributions, some generalization may be possible because of the substantial overall reported data sets from varying ecological environments. The meta‐analysis by Soininen et al. (2012) was one such attempt at a generalization with no consistent result, but rather a mixture of negative, positive, or insignificant correlations between richness and evenness. Such lack of consistent results or association between richness and evenness as diversity components also highlights the important point that the frequently used richness by itself is an incomplete measure of diversity.

One way to use a more general data base than those based on particular ecological systems for exploring potential richness‐evenness relationships is the use of randomly generated relative abundance distributions $P_{n}=\left(p_{1},\ldots ,p_{n}\right)$ where the richness *n* and each $p_{i}\;\left(i=1,\ldots ,n\right)$ are obtained by random number generation. Random sample data can then be used, for example, to test whether any statistically significant correlation exists between richness and evenness. This approach is one of the objectives of the present article. Such random sample data will also be used to assess the potential associations between different diversity measures and between different evenness measures.

Another objective of this article is to determine analytically the relative effects of richness versus evenness on the values of specific diversity measures. Four well‐known diversity measures will be considered, including Simpson's index and the entropy index.

Besides analyses of potential relationships between richness and evenness based on data for which diversity is also a variable, a further objective of this article is to present a method for considering the richness‐evenness relationship for fixed diversity. With richness and evenness being components of diversity, one could consider the tradeoff between those two components for any given value of the diversity. Thus, for any given relative abundance distribution $P_{n}=\left(p_{1},\ldots ,p_{n}\right)$ and some diversity index with the value $D\left(P_{n}\right)$, one could consider other distributions $P_{m}$ with the same diversity value, that is, $D\left(P_{n}\right)=D\left(P_{m}\right)$, but with different richness $\left(m\neq n\right)$ and different evenness.

Such richness‐evenness tradeoff relationships or graphical curves for fixed diversity will necessarily depend on the diversity measure being used as will be exemplified and illustrated in this article. Although intuitively rather simple to comprehend as a general concept, rigorous analysis and description of the relationship between richness and evenness for a given value of a diversity measure will require some mathematical formulations. Those developments will also emphasize the validity of the formulations. Real biological data will be used as numerical examples.

## 2 DIVERSITY, RICHNESS, AND EVENNESS

### 2.1 Definitions

In the most general terms, consider the case of *n* mutually exclusive and exhaustive categories with the respective probabilities or proportions $p_{1},p_{2},\ldots ,p_{n}$ with each $p_{i}\geq 0$ and $\sum_{i=1}^{n}p_{i}=1$. In biology or ecology, $P_{n}=\left(p_{1},\ldots ,p_{n}\right)$ becomes the abundance distribution for *n* different species, with *n* typically being referred to as the species richness. For a generic diversity measure *D*, the value $E\left(P_{n}\right)$ of the corresponding evenness index *E* for the distribution $P_{n}$ can be defined as the following normalized form of *D* (Kvålseth, 2015):

(1) $$E\left(P_{n}\right)=D^{*}\left(P_{n}\right)=\frac{D\left(P_{n}\right)-D\left(P_{n}^{0}\right)}{D\left(P_{n}^{1}\right)-D\left(P_{n}^{0}\right)}\in \left[0,1\right]$$

involving the degenerate and uniform distributions.

(2) $$P_{n}^{0}=\left(1,0,\ldots ,0\right),P_{n}^{1}=\left(1/n,\ldots ,1/n\right)$$

The term $D\left(P_{n}^{1}\right)$ in (1) becomes a function of the richness *n*.

In its most general form, the diversity value $D\left(P_{n}\right)$ as a function of $D^{*}\left(P_{n}\right)$ and *n* can be expressed from (1) as

(3) $$D\left(P_{n}\right)=\left[D\left(P_{n}^{1}\right)-D\left(P_{n}^{0}\right)\right]D^{*}\left(P_{n}\right)+D\left(P_{n}^{0}\right)$$

For the apparently most popular diversity indices, (3) reduces to the following expressions: for Simpson's index (Simpson, 1949),

(4) $$D_{S}\left(P_{n}\right)=1-\sum_{i=1}^{n}p_{i}^{2}=\left(1-1/n\right)D_{S}^{*}\left(P_{n}\right)$$

for the entropy due to Shannon (1948),

(5) $$H\left(P_{n}\right)=-\sum_{i=1}^{n}p_{i}\log p_{i}=\left(\log n\right)H^{*}\left(P_{n}\right)$$

for the second form of Simpson's index,

(6) $$D_{S2}\left(P_{n}\right)={\left(\sum_{i=1}^{n}p_{i}^{2}\right)}^{-1}=\left(n-1\right)D_{S2}^{*}\left(P_{n}\right)+1$$

and, for the exponential form of the entropy in (5), apparently first proposed by Sheldon (1969),

(7) $$H_{2}\left(P_{n}\right)=\exp\left(H\left(P_{n}\right)\right)=\left(n-1\right)H_{2}^{*}\left(P_{n}\right)+1$$

While the above expressions involve the general distribution $P_{n}=\left(p_{1},\ldots ,p_{n}\right)$, another special distribution that will be useful in the subsequent analysis is the *lambda distribution* introduced by Kvålseth (2011) and defined as follows:

(8) $$P_{n}^{\lambda }=\left(1-\lambda +\frac{\lambda }{n},\frac{\lambda }{n},\ldots ,\frac{\lambda }{n}\right),\lambda \in \left[0,1\right]$$

where λ is an evenness parameter. This $P_{n}^{\lambda }$ is a so‐called mixture distribution, being the weighted mean of the extreme distributions $P_{n}^{0}$ and $P_{n}^{1}$ in (2), that is,

(9) $$P_{n}^{\lambda }=\left(1-\lambda \right)P_{n}^{0}+\lambda P_{n}^{1}$$

For the analysis of some diversity index *D*, the utility of $P_{n}^{\lambda }$ comes from the fact that for any $P_{n}$,

(10) $$D\left(P_{n}\right)=D\left(P_{n}^{\lambda }\right)\;\text{for}\;a\;\text{unique}\;\lambda$$

as exemplified next.

In terms of the notation used in this article, it should be noted that the strictly mathematically correct notation would be to use *D* to denote a diversity function and $D\left(P_{n}\right)$ to denote its value for the distribution $P_{n}$ as used above. However, for the sake of simplicity and where there is no chance of ambiguity, *D* may sometimes be used both as a function and its numerical value. The same comment applies to other summary measures used in the article.

### 2.2 Value validity

For any measure of evenness, as with summary measures in general, it is essential that all values of a measure provide true, realistic, or valid representations of the attribute being measured, that is, the evenness characteristic. The conditions for such *value‐validity property*, first introduced by Kvålseth (2014), have been discussed in detail for evenness indices by Kvålseth (2015).

As a brief outline here, the value‐validity condition for the normalized diversity index $D^{*}$ as a measure of evenness can be derived as follows. Consider first the distribution $P_{n}^{\lambda }$ in (8) and its extreme members in (2) as points (vectors) in *n*‐dimensional Euclidean space, with $D^{*}\left(P_{n}^{0}\right)=0$ and $D^{*}\left(P_{n}^{1}\right)=1$. Then, in terms of the Euclidean distance function *d*, the evenness parameter λ can be expressed in terms of metric distances as follows:

(11) $$d^{*}\left(P_{n}^{\lambda }\right)=\frac{d\left(P_{n}^{0},P_{n}^{1}\right)-d\left(P_{n}^{\lambda },P_{n}^{1}\right)}{d\left(P_{n}^{0},P_{n}^{1}\right)}=\lambda$$

where $d\left(P_{n}^{0},P_{n}^{1}\right)=\max_{P_{n}^{\lambda }}d\left(P_{n}^{\lambda },P_{n}^{1}\right)$. That is, λ equals the relative extent to which the Euclidean distance between $P_{n}^{\lambda }$ and $P_{n}^{1}$ is less than its maximum distance. The value‐validity condition on the diversity *D* requires that

(12) $$D^{*}\left(P_{n}^{\lambda }\right)=d^{*}\left(P_{n}^{\lambda }\right)=\lambda$$

or as an approximation. For the general distribution $P_{n}=\left(p_{1},\ldots ,p_{n}\right)$ and from (10), the condition in (12) becomes

(13) $$D^{*}\left(P_{n}\right)=d^{*}\left(P_{n}\right)$$

or approximately so, with $P_{n}$ substituted for $P_{n}^{\lambda }$ in (11).

Therefore, according to (12) and (13), $D^{*}\left(P_{n}^{\lambda }\right)$ and $D^{*}\left(P_{n}\right)$ measure the relative proximity of $P_{n}^{\lambda }$ and $P_{n}$ to the complete evenness distribution $P_{n}^{1}$ based on Euclidean distances. For example, for the simple distribution $P_{2}^{1/2}=\left(0.75,0.25\right)=\left(\frac{1}{2}\right)P_{2}^{0}+\left(\frac{1}{2}\right)P_{2}^{1}$, (12) requires that $D^{*}\left(P_{2}^{1/2}\right)=1/2$, which is clearly a most logical result. Nevertheless, none of the diversity indices in (4)–(7) satisfies this condition (Kvålseth, 2015). However, as discussed below, those indices can be corrected so as to comply.

## 3 RANDOM SAMPLE RESULTS

The wide variety of reported biological studies of the potential relationships between richness and evenness have involved various types of species and environments. Consequently, the results from such studies apply to those specific situations and may not be generalizable to other situations. In order to explore some more general data, distributions $P_{n}=\left(p_{1},\ldots ,p_{n}\right)$ were generated randomly using the computer algorithm described in Kvålseth (2015). Thus, the richness $n\in \left[2,100\right]$ and the value of each $p_{i}$ were generated as random numbers within given intervals. The results are summarized in Table 1.

**TABLE 1:** Sample values from randomly generated distributions $P_{n}=\left(p_{1},\ldots ,p_{n}\right)$ of the measures $D_{S}$ and $D_{S}^{*}$ defined in (4), $D_{S2}$ and $D_{S2}^{*}$ in (6), H and $H^{*}$ in (5), and $H_{2}$ and $H_{2}^{*}$ in (7).

Data set	*n*	$D_{S}$	$D_{S}^{*}$	$D_{S2}$	$D_{S2}^{*}$	H	$H^{*}$	$H_{2}$	$H_{2}^{*}$
1	5	0.62	0.78	2.62	0.41	1.20	0.75	3.32	0.58
2	30	0.50	0.52	2.00	0.03	1.31	0.39	3.71	0.09
3	62	0.92	0.94	12.63	0.19	3.55	0.86	34.81	0.55
4	70	0.24	0.24	1.31	0.00	0.85	0.20	2.34	0.02
5	78	0.88	0.89	8.02	0.09	3.48	0.80	32.46	0.41
6	78	0.81	0.82	5.33	0.06	3.08	0.71	21.76	0.27
7	88	0.97	0.98	38.77	0.43	4.20	0.94	66.69	0.76
8	10	0.73	0.81	3.71	0.30	1.72	0.75	5.58	0.51
9	39	0.87	0.89	7.56	0.17	2.91	0.79	18.36	0.46
10	26	0.96	1.00	24.50	0.94	3.23	0.99	25.28	0.97
11	79	0.90	0.91	10.20	0.12	3.24	0.74	25.53	0.31
12	36	0.11	0.11	1.13	0.00	0.42	0.12	1.52	0.01
13	90	0.91	0.92	11.21	0.11	3.51	0.78	33.45	0.36
14	19	0.84	0.89	6.43	0.30	2.48	0.84	11.94	0.61
15	75	0.90	0.91	9.82	0.12	3.49	0.81	32.79	0.43
16	43	0.93	0.95	14.84	0.33	3.41	0.91	30.27	0.70
17	71	0.91	0.92	10.79	0.14	3.44	0.81	31.19	0.43
18	19	0.90	0.95	9.55	0.48	2.68	0.91	14.59	0.76
19	42	0.62	0.64	2.66	0.04	2.09	0.56	8.08	0.17
20	17	0.67	0.71	3.02	0.13	1.86	0.66	6.42	0.34
21	11	0.01	0.01	1.01	0.00	0.02	0.01	1.02	0.00
22	95	0.96	0.97	22.85	0.23	4.16	0.91	64.07	0.67
23	91	0.02	0.02	1.02	0.00	0.09	0.02	1.09	0.00
24	68	0.96	0.97	22.32	0.32	3.90	0.92	49.40	0.72
25	82	0.75	0.76	4.02	0.04	2.81	0.64	16.61	0.19
26	12	0.86	0.94	6.96	0.54	2.26	0.91	9.58	0.78
27	20	0.88	0.93	8.31	0.38	2.61	0.87	13.60	0.66
28	17	0.38	0.40	1.60	0.04	1.08	0.38	2.94	0.12
29	21	0.74	0.78	3.83	0.14	1.94	0.64	6.96	0.30
30	91	0.30	0.30	1.42	0.00	1.16	0.26	3.19	0.02

While some of the measures included in Table 1 will be defined in subsequent derivations, one conclusion that can be drawn from the data for the diversity measures in (4)–(7) is that no apparent relationship seems to exist between richness and evenness. Based on the statistical results for the Pearson correlation coefficient (*r*) for Data Sets 1–4 in Table 2, the absolute values of the *t*‐statistic are all less than the critical value $t_{28,\alpha /2}=2.048$ for the significance level α=0.05 so that the null‐hypothesis of zero (population) correlation cannot be rejected. Similarly, from the four *p*‐values in Table 2, there is sufficient evidence to conclude that the correlation between richness and evenness for each of the diversity measures in (4)–(7) is not significantly different from zero. Of course, zero correlation does not necessarily imply statistical independence since other than linear relationships may exist between evenness and richness. However, from the data in Table 1, no other potential relationship seems plausible.

**TABLE 2:** Pearson's correlation coefficients (*r*) and the *t*‐statistics for the null hypothesis that the population correlation ρ=0 (versus ρ≠0) as well as the *p*‐values based on the data in Table 1.

Data set	*r*	*r*‐value	*t*‐statistic	*p*‐value
1	$r\left(D_{S}^{*},n\right)$	.04	0.21	.84
2	$r\left(D_{S2}^{*},n\right)$	−.35	−1.97	.06
3	$r\left(H^{*},n\right)$	−.01	−0.05	.96
4	$r\left(H_{2}^{*},n\right)$	−.18	−0.97	.34
5	$r\left(D_{S}^{*},D_{S2}^{*}\right)$	.60	3.97	.0005
6	$r\left(H^{*},H_{2}^{*}\right)$	.90	10.93	.00001
7	$r\left(D_{S}^{*},H^{*}\right)$	.99	37.14	.00001
8	$r\left(D_{S2}^{*},H_{2}^{*}\right)$	.89	10.33	.00001
9	$r\left(D_{S},H\right)$	.93	13.39	.00001
10	$r\left(D_{S},D_{S2}\right)$	.64	4.41	.000 1
11	$r\left(H,H_{2}\right)$	.88	9.80	.00001
12	$r\left(D_{S2},H_{2}\right)$	.89	10.33	.00001

It is important to point out that even though the values of the different evenness indices are found to be highly correlated as shown in Table 2 (Data Sets 5–8), their individual values can differ greatly as seen from the results in Table 1. For example, when comparing $D_{S}^{*}$ and $D_{S2}^{*}$, $\text{RMSE}\left(D_{S}^{*},D_{S2}^{*}\right)={\left[\sum_{i=1}^{30}{\left(D_{\mathrm{Si}}^{*}-D_{S2i}^{*}\right)}^{2}/30\right]}^{1/2}=0.58$ and $\text{RMSE}\left(H^{*},H_{2}^{*}\right)=0.28$. Similarly differing results are seen when comparing the diversity values in Table 1. Although the correlation coefficients in Table 2 (Data Sets 9–12) are quite impressive, different diversity measures can produce substantially different results for the same distributions $P_{n}=\left(p_{1},\ldots ,p_{n}\right)$. For example, for $D_{S2}$ in (6) and $H_{2}$ in (7), which both take on values within the same [1, *n*]‐interval, it is found that $\text{RMSE}\left(D_{S2},H_{2}\right)=15.18$. Comparative results such as these give reason for concern when using even some of the most popular measures of diversity and evenness. However, such concern is alleviated in the subsequent richness‐evenness analysis, where value‐validity corrections are being applied to the evenness indices.

## 4 RICHNESS‐EVENNESS TRADEOFF

### 4.1 General tradeoff formulation

While the preceding analysis is concerned with potential relationships between richness and evenness, or lack thereof, when based on varying distributions $P_{n}=\left(p_{1},\ldots ,p_{n}\right)$, consider now the following question: what are the relative effects of richness and evenness on a given diversity measure for any individual distribution $P_{n}$? That is, what is the tradeoff between richness and evenness for any given diversity value $D\left(P_{n}\right)$? Or, how can different combinations of richness and evenness produce the same given $D\left(P_{n}\right)$‐value?

The most obvious answer to this question would seem to lie in the definition in (3). Thus, for any given $D\left(P_{n}\right)$, one could simply replace the $P_{n}$ on the right side of (3) with any other distribution $P_{m}=\left(p_{1},\ldots ,p_{m}\right)$ such that $D\left(P_{m}\right)=D\left(P_{n}\right)$ and then solve the resulting equation for $D^{*}\left(P_{m}\right)$ as a function of *m*. However, since the evenness indices in (4)–(7) do not meet the value‐validity condition in (12) (Kvålseth, 2015), an alternative approach should be considered.

In terms of the lambda distribution $P_{n}^{\lambda }$ in (8) and a generic diversity measure *D*, the relationship in (10) can be generalized such that for any given $P_{n}$, the given value $D\left(P_{n}\right)$ can equal $D\left(P_{m}^{\lambda }\right)$ for various unique $\left(m,\lambda \right)$‐pairs. There would necessarily be a certain restriction on *m* depending upon the value $D\left(P_{n}\right)$. For a chosen *m*, the $D\left(P_{m}^{\lambda }\right)$ becomes a function of λ that can be solved for λ as the proper evenness value. The resulting λ may conveniently be denoted by $D_{C}^{*}\left(P_{m}\right)$, or simply $D_{C}^{*}$, to indicate that the normalized $D^{*}$ has been corrected to satisfy the value‐validity condition in (12).

This procedure may be summarized as follows: for any given $D\left(P_{n}\right)$,

(14) $$D\left(P_{n}\right)=D\left(P_{m}^{\lambda }\right)⟹\lambda =D_{C}^{*}=f\left[D\left(P_{n}\right),m\right]$$

where *f* is a function of $D\left(P_{n}\right)$ and *m*. The formulation in (14) holds for any $\lambda \in \left[0,1\right]$ and all *m* subject to a restriction $m\geq m_{0}\left[D\left(P_{n}\right)\right]$ that depends on the value $D\left(P_{n}\right)$ and consequently on the form of the diversity measure as will be exemplified next for the diversity measures in (4)–(7). By varying *m* and $D_{C}^{*}$ in (14), the results can also be represented graphically as *richness‐evenness curves*, or *R‐E curves*, for potentially interesting and useful diversity analysis.

### 4.2 Simpson's measures

For Simpson's index in (4), it follows from (14) and $P_{m}^{\lambda }$ defined in (9) that

(15) $$D_{S}\left(P_{n}\right)=D_{S}\left(P_{m}^{\lambda }\right)=\left(1-1/m\right)\lambda \left(2-\lambda \right)$$

which, solved for λ as the value‐validity corrected evenness $D_{\mathrm{SC}}^{*}$, gives

(16) $$\lambda =D_{\mathrm{SC}}^{*}=1-\sqrt{1-\frac{mD_{S}\left(P_{n}\right)}{m-1}}$$

This relationship holds for all $D_{\mathrm{SC}}^{*}\in \left[0,1\right]$ and $m\geq 1/\left(1-D_{S}\left(P_{n}\right)\right)$ (for the square root to be defined).

As a simple example, consider the distribution $P_{5}=\left(0.40,0.30,0.15,0.10,0.05\right)$ for which $D_{S}\left(P_{5}\right)=0.72$ so that from (16),

$$D_{\mathrm{SC}}^{*}=1-\sqrt{1-\frac{m\left(0.72\right)}{m-1}}$$

as the tradeoff relationship between the richness *m* and evenness $D_{\mathrm{SC}}^{*}$ for the fixed diversity value $D_{S}\left(P_{5}\right)=0.72$. The resulting graph of $D_{\mathrm{SC}}^{*}$ as a function of *m* for $m\geq 1/\left(1-0.72\right)=3.57$ (or 4) becomes the *R‐E* (tradeoff) curve for $D_{S}\left(P_{5}\right)=0.72$. By choosing, for instance, the richness value m=10, the corresponding evenness value (point) along the *R‐E* curve would be $D_{\mathrm{SC}}^{*}=1-\sqrt{1-10\left(0.72\right)/9}=0.55$.

Other diversity measures that are strictly increasing functions of $D_{S}\left(P_{n}\right)$ will necessarily have the same richness‐evenness curve as that of $D_{S}\left(P_{n}\right)$ for the same $P_{n}$. Such diversity measures include $D_{S2}\left(P_{n}\right)$ in (6), the statistical odds measure $D_{S2}\left(P_{n}\right)-1$ by Kvålseth (1991), $-\log\sum_{i=1}^{n}p_{i}^{2}$ proposed by Pielou (1977), $\left(1-\sqrt{\sum_{i=1}^{n}p_{i}^{2}}\right)/\left(1-\sqrt{1/N}\right)$ by McIntosh (1967) where *N* is the sample size, and $1-\sqrt{\sum_{i=1}^{n}p_{i}^{2}}$ proposed by Junge (1994). As an example of this fact, consider the form $D_{S2}\left(P_{n}\right)$ of Simpson's index in (6) for which

$$D_{S2}\left(P_{n}\right)=D_{S2}\left(P_{m}^{\lambda }\right)={\left[1-\left(1-\frac{1}{m}\right)\lambda \left(2-\lambda \right)\right]}^{-1}$$

with $P_{m}^{\lambda }$ defined in (8). Solving this expression for λ as the value‐validity corrected evenness $\lambda =D_{S2C}^{*}$, it is readily seen that $D_{S2C}^{*}$ as a function of $D_{S2}\left(P_{n}\right)$ and *m* is exactly the same as that of (16) with $D_{S}\left(P_{n}\right)=1-{\left(D_{S2}\left(P_{n}\right)\right)}^{-1}$.

**FIGURE 1:**
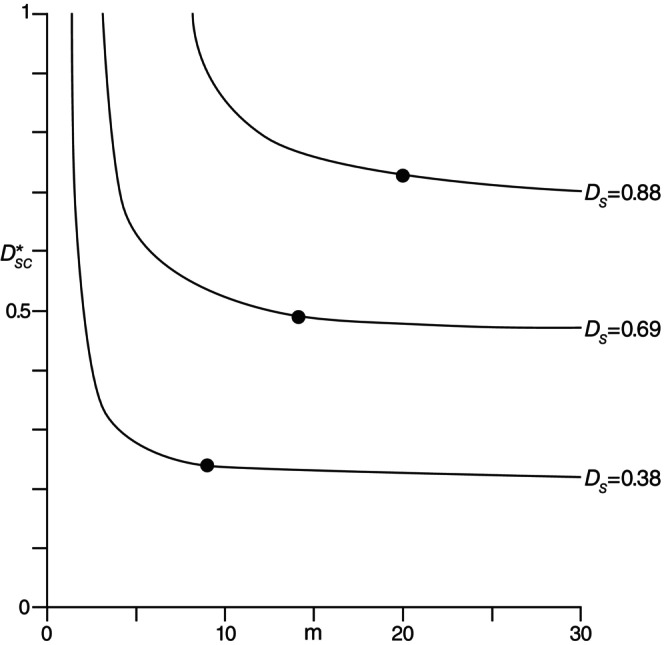
Richness‐evenness curves for Simpson's index from (16) for three different abundance distributions (see text).

Examples of such curves are given in Figure 1 based on real data from Magurran (2004), with $D_{S}\left(P_{9}\right)=0.38$ (p. 243, “Unburned forest”), $D_{S}\left(P_{14}\right)=0.69$ (p. 243, “Burned chaparral”), and $D_{S}\left(P_{20}\right)=0.88$ (pp. 237–238, “Derrycunnitry oakwood”). Those species abundance distributions cover a wide range of diversity values. These curves cover richness‐values for m≤30, although the asymptotic values of $D_{\mathrm{SC}}^{*}\left(P_{m}\right)$ are seen from (16) to be $1-\sqrt{1-D_{S}\left(P_{n}\right)}$ as m→∞. The curves are presented as being continuous, but they are obviously most meaningful for integer values of *m*.

### 4.3 Entropy measures

In order to obtain the *R‐E* curve for the commonly used entropy $H\left(P_{n}\right)$ in (5), it becomes immediately clear that setting $H\left(P_{n}\right)=H\left(P_{m}^{\lambda }\right)$ for any given abundance distribution $P_{n}$ and the lambda distribution $P_{m}^{\lambda }$ in (8) cannot be readily solved for $\lambda =H_{C}^{*}$ as required by (14). One could, of course, use a search procedure to obtain all combinations for *m* and λ such that $H\left(P_{n}\right)=H\left(P_{m}^{\lambda }\right)$ for any given $H\left(P_{n}\right)$. Alternatively, as a more convenient and practical approach, good approximate results can be obtained from the following formulation by Kvålseth (2014):

(17) $$\lambda \approx H_{C}^{*}=1-{\left[1-{\left(\frac{H\left(P_{n}\right)}{\log m}\right)}^{4/3}\right]}^{\alpha },\alpha =\left(\frac{1}{2}\right){\left(m-1\right)}^{1/9}$$

All combinations of *m* and $H_{C}^{*}$ in (17) have the same entropy value $H\left(P_{n}\right)$, with $m\geq \exp\left[H\left(P_{n}\right)\right]$ and $H_{C}^{*}$ being the (approximate) value‐validity corrected evenness index for $H\left(P_{n}\right)$.

As a computational example, consider $P_{5}=\left(0.40,0.30,0.15,0.10,0.05\right)$ as used above for $D_{\mathrm{SC}}^{*}$. With $H\left(P_{5}\right)=1.39$ and for m=n=5, it follows from (17) that α=0.58 and hence $H_{C}^{*}\left(P_{5}\right)=0.63$. By choosing, say, m=10, it is found from (17) that α=0.64 and hence $H_{C}^{*}\left(P_{10}\right)=0.37$. The comparable values for $D_{\mathrm{SC}}^{*}$ from (16) are $D_{\mathrm{SC}}^{*}\left(P_{m}\right)=0.68\;\text{and}\;0.55$, indicating that individual values of $D_{\mathrm{SC}}^{*}$ and $H_{C}^{*}$ can differ considerably. As with alternative diversity measures that are strictly increasing functions of $D_{S}\left(P_{n}\right)$, those that are strictly increasing functions of $H\left(P_{n}\right)$ such as $H_{2}\left(P_{n}\right)$ in (7) will have the same *R‐E* curve as that of $H\left(P_{n}\right)$ for any given $P_{n}$.

**FIGURE 2:**
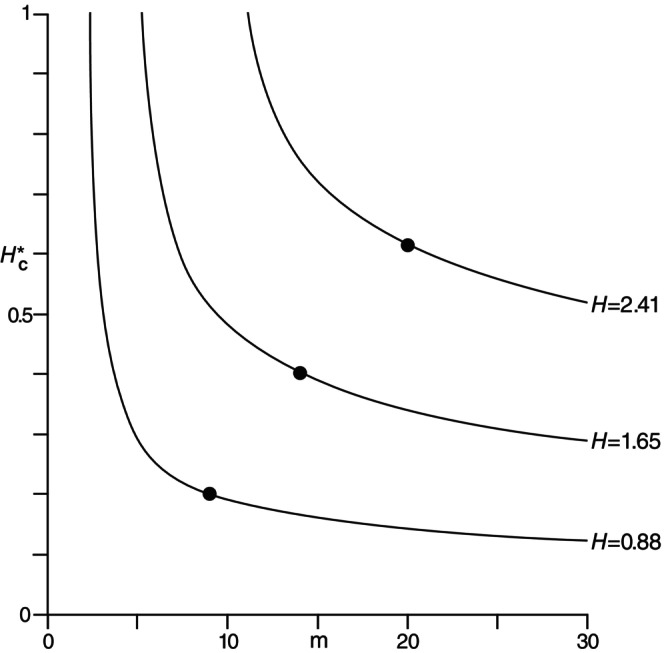
Richness‐evenness curves for the entropy index from (17) for the same three different abundance distributions as those used in Figure 1 (see text).

As examples of *R‐E* curves for $H\left(P_{n}\right)$ involving real biological data, those used for $D_{S}\left(P_{n}\right)$ and represented by Figure 1 will also be used for $H\left(P_{n}\right)$ with the respective values $H\left(P_{9}\right)=0.88$, $H\left(P_{14}\right)=1.65$, and $H\left(P_{20}\right)=2.41$. The three curves are given in Figure 2.

### 4.4 Comments on the *R‐E* curves

The general richness‐evenness (*R‐E*) curve is based on evenness $D_{C}^{*}\left(P_{m}\right)$, or simply $D_{C}^{*}$, as a function of the richness *m* for some given (fixed) entropy value $D\left(P_{n}\right)$ as expressed in (14). Some general properties of an *R‐E* curve are as follows. First, each point (*m*, $D_{C}^{*}$) on the curve has the same entropy value $D\left(P_{n}\right)$. In order to produce such a curve, which is somewhat analogous in shape to the *indifference curve* used in economics (e.g., Varian, 2010), only the diversity value $D\left(P_{n}\right)$ and the form of *D* need to be known. Second, the *R‐E* curve is convex (i.e., bowed toward the origin). Third, the *R‐E* curve has a negative slope. Fourth, since the function *f* in (14) is assumed to be a single‐valued function, *R‐E* curves cannot intersect. Fifth, curves with increasing distance from the origin represent increasing diversity.

These properties are all rather evident from the real data curves in Figures 1 and 2. Although the general shapes of the curves are quite similar for Simpson's $D_{S}$ in (4) and the entropy *H* in (5), their specific details clearly differ considerably. Even though one of the properties of *R‐E* curves is that they cannot intersect for the same diversity measure, they can for different diversity measures as is apparent from Figures 1 and 2. Thus, for instance, the middle curves for $D_{S}\left(P_{14}\right)=0.69$ and $H\left(P_{14}\right)=1.65$ are seen to intercept approximately at the point when m=8 and $D_{\mathrm{SC}}^{*}\left(P_{8}\right)=H_{C}^{*}\left(P_{8}\right)=0.55$. At this crossover point, the $D_{S}\left(P_{14}\right)$ and $H\left(P_{14}\right)$ have the same richness and evenness components, otherwise they differ for this $P_{14}$‐distribution. For m>8, the rate of change of evenness with increasing *m* is clearly greater for $H\left(P_{14}\right)$ than for $D_{S}\left(P_{14}\right)$, whereas for m<8, this rate of change is more comparable.

One of the most striking characteristics common to all of these *R‐E* curves is the rather dramatic negative slopes for the smaller *m* values where the evenness values approach unity as *m* approaches the respective lower limits of $1/\left(1-D_{S}\left(P_{n}\right)\right)$ and $\exp\left(H\left(P_{n}\right)\right)$. That is, for a given or constant diversity value $D\left(P_{n}\right)$ and for any other distribution $P_{m}=\left(p_{1},\ldots ,p_{m}\right)$ such that $D\left(P_{m}\right)=D\left(P_{n}\right)$, the evenness is most sensitive to changes in *m* for small *m*‐values.

The fact that the actual richness m=n and evenness $D_{C}^{*}\left(P_{n}\right)$ is a single point on an *R‐E* curve representing a given diversity value $D\left(P_{n}\right)$, such as the points identified in Figures 1 and 2, indicates the considerable amount of potentially interesting and useful information available in such a curve. The use of such information depends, of course, on the particular interest of a user or researcher in any given situation. With all points along an *R‐E* curve having the same diversity, perhaps the single most useful aspect lies in the ability to see how the diversity components, richness and evenness, can be traded off and still produce the same diversity as that of the original data set. Far from being independent, richness depends entirely on evenness, or vice versa, for any given *R‐E* curve.

While an individual *R‐E* curve can provide information about the potential richness‐evenness tradeoff characteristic for a particular data set or diversity value, different *R‐E* curves can also be used for comparing the diversity values for different data sets or distributions $P_{n}$ when controlling for either richness or evenness. As an example, consider the three curves in Figure 1 and control for *m* by looking at the point on each curve with say, m=20. For the top curve, this point corresponds to the diversity $D_{S}\left(P_{20}\right)=0.88$ for the real $P_{20}$ from Magurran (2004, pp. 237–238). Comparing the $D_{\mathrm{SC}}^{*}$ values for the three points with m=20 shows how the differences between the three diversity values (0.38, 0.69, and 0.88) are due to the equal differences between the respective evenness values of 0.23, 0.48, and 0.73 as seen from Figure 1 or computed from (16).

As stated at the beginning of Section 4, the tradeoff between richness and evenness for any given diversity value $D\left(P_{n}\right)$ could also be considered in terms of (3) as

$$D\left(P_{m}\right)=\left[D\left(P_{m}^{1}\right)-D\left(P_{m}^{0}\right)\right]D^{*}\left(P_{m}\right)+D\left(P_{m}^{0}\right)=D\left(P_{n}\right)$$

and then simply determine $D^{*}\left(P_{m}\right)$ as a function of *m*. The resulting richness‐evenness curves would at least resemble in form those of $D_{C}^{*}$ versus *m* as presented above and would be entirely appropriate if $D^{*}\left(P_{m}\right)$ is assumed to be an acceptable evenness measure. However, since this assumption can indeed be challenged for various diversity measures (Kvålseth, 2015), the value‐validity corrected evenness measures $D_{C}^{*}\left(P_{m}\right)$ are used as a more appropriate representation. Also, while the value‐validity requirement is discussed quite concisely in this article, more detailed explanations are given by Kvålseth (2011, 2014, 2015).

### 4.5 Relative effects of richness and evenness

It is rather evident from the data in Table 1 that evenness generally contributes more toward diversity than does richness or that diversity is more sensitive to changes in evenness than to changes in richness. Since the range of variation of those two characteristics differs greatly, one reasonable way to compare their effects on diversity is to consider relative changes in diversity related to relative changes in evenness and richness by a method analogous to *partial elasticity* widely used in economics (e.g., Varian, 2010, pp. 274–291).

Thus, in terms of relative changes and partial derivatives, one can define the following measure for $D_{S}$ in (4):

(18) $$ED_{S}^{*}=\lim_{∆D_{S}^{*}\to 0}\frac{∆D_{S}/D_{S}}{∆D_{S}^{*}/D_{S}^{*}}=\left(\frac{\partial D_{S}}{\partial D_{S}^{*}}\right)\left(\frac{D_{S}^{*}}{D_{S}}\right)$$

which measures the sensitivity of $D_{S}$ to a (relative) change in $D_{S}^{*}$ while keeping richness *n* fixed. Also, by treating *n* as a continuous variable for purely mathematical purpose and by similarly defining En as in (18), the following result is obtained:

(19) $$ED_{S}^{*}=n\left(n-1\right)\mathrm{En}$$

That is, for n>2, $D_{S}$ is more sensitive to changes in evenness than to changes in richness (*n*), especially for large *n*.

Similarly, for the entropy measure in (5) and the equivalent relative change expressions for $H^{*}$ and *n* to that of (18), it is determined that

(20) $$EH^{*}=\left(\log n\right)\mathrm{En}$$

which shows that, for n>2, the entropy index is more sensitive to changes in $H^{*}$ than to changes in *n*. When comparing (19) and (20), it would seem that such differences between evenness and richness in their effects on diversity are more pronounced in the case of $D_{S}$ in (4) than of *H* in (5).

In terms of the same definition as in (18), the following result is obtained for $D_{S2}$ in (6) and $H_{2}$ in (7):

(21) $$ED_{S2}^{*}=\left(1-\frac{1}{n}\right)\mathrm{En},EH_{2}^{*}=\left(1-\frac{1}{n}\right)\mathrm{En}$$

The inference from (21) is that both diversity measures $D_{S2}$ and $H_{2}$ tend to be somewhat less sensitive to changes in evenness than to changes in richness, but only marginally so when *n* is large. Interestingly, this finding is opposite those in (19) and (20).

## 5 CONCLUSION

There are three main findings from this analysis. First, when considering the results from randomly generated abundance distributions $P_{n}=\left(p_{1},\ldots ,p_{n}\right)$ for the four well‐known diversity measures in (4)–(7), the conclusion is that there is no statistically significant correlation between the richness and evenness components of those diversity measures. Second, in spite of such lack of association between richness and evenness across abundance distributions, one can analyze the relationship between richness and evenness for any given $P_{n}$ and any diversity index by means of the *richness‐evenness curve* introduced above. Third, when considering relative changes in the values of a diversity measure as the result of a relative change in richness (with evenness kept fixed) versus a relative change in evenness (with richness kept fixed), $D_{S}$ in (4)and *H* in (5) are found to be more sensitive to changes in evenness than to richness, with the reverse finding for $D_{S2}$ in (6) and $H_{2}$ in (7).

The richness‐evenness (*R‐E*) curve provides a new tool for analyzing diversity. Such a curve can provide interesting and useful information about the potential tradeoff between the richness and evenness components of a given value of any diversity measure. It also has potential utility when comparing the diversity values for different abundance distributions and when comparing the behavior of different diversity measures.

## AUTHOR CONTRIBUTIONS

**Tarald O. Kvålseth:** Conceptualization (equal); data curation (equal); formal analysis (equal); funding acquisition (equal); investigation (equal); methodology (equal); project administration (equal); resources (equal); software (equal); supervision (equal); validation (equal); visualization (equal); writing – original draft (equal); writing – review and editing (equal).

## FUNDING INFORMATION

None.

## CONFLICT OF INTEREST STATEMENT

The author declares no conflict of interest.

## Data Availability

All relevant data are included in the manuscript.
